# Dual band metamaterial perfect absorber based on artificial dielectric “molecules”

**DOI:** 10.1038/srep28906

**Published:** 2016-07-13

**Authors:** Xiaoming Liu, Chuwen Lan, Bo Li, Qian Zhao, Ji Zhou

**Affiliations:** 1 State Key Laboratory of New Ceramics and Fine Processing, School of Materials Science and Engineering, Tsinghua University, Beijing 100084, China; 2 State Key Lab of Tribology, Department of Precision Instruments and Mechanology, Tsinghua University, Beijing 100084, China

## Abstract

Dual band metamaterial perfect absorbers with two absorption bands are highly desirable because of their potential application areas such as detectors, transceiver system, and spectroscopic imagers. However, most of these dual band metamaterial absorbers proposed were based on resonances of metal patterns. Here, we numerically and experimentally demonstrate a dual band metamaterial perfect absorber composed of artificial dielectric “molecules” with high symmetry. The artificial dielectric “molecule” consists of four “atoms” of two different sizes corresponding to two absorption bands with near unity absorptivity. Numerical and experimental absorptivity verify that the dual-band metamaterial absorber is polarization insensitive and can operate in wide-angle incidence.

Crystal is formed by periodically arranged atoms, ions or molecules bonding together by attractive and repulsive forces. Similar to this natural material, metamaterial is composed of periodically arranged artificial “atoms” or “molecules” with predesigned dimensions. However, the exotic electromagnetic effects such as negative index of refraction^1^,^2^,^3^,^4^,^5^,^6^,^7^,^8^, superlens^9^,^10^,^11^,^12^,^13^, cloak^14^,^15^,^16^,^17^,^18^ and perfect absorption^19^,^20^,^21^,^22^,^23^,^24^,^25^,^26^,^27^,^28^,^29^,^30^,^31^,^32^ make metamaterials more attractive than natural materials. Metamaterial perfect absorbers composed of structured subwavelength artificial “atoms” can be considered as homogeneous materials whose effective electric permittivity *ε*(*ω*) and magnetic permeability *μ*(*ω*) can be changed separately at resonant frequency by altering the size or shape of artificial “atoms”. According to the relationship among absorptivity *A*, reflectivity *R,* and transmissivity *T*, *A* = 1-*R*-*T*, near unity absorptivity can be obtained at predesigned frequency when both reflectivity *R* and transmissivity *T* are minimized. *T* is zero across the entire frequency range when we add a metallic ground plane thicker than the penetration depth. Therefore, the critical issue to achieve near unity absorptivity is to make reflectivity *R* = 0. When the incident electromagnetic wave is normal to the metamaterial absorber, the reflectivity *R* is as follows:


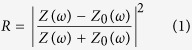


where 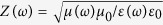 is the impedance of the metamaterial absorber and 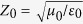 is the impedance of free space. If we want reflectivity *R* = 0, the impedance match condition must be met: 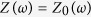, leading to *ε*(*ω*) = *μ*(*ω*). Metamaterials make it possible to tune the effective electric permittivity *ε*(*ω*) and magnetic permeability *μ*(*ω*) simultaneously. Therefore, metamaterial absorbers can be designed to perform near unity absorption at certain frequency by impedance-matched to free space *ε*(*ω*) = *μ*(*ω*). Due to the interesting absorption mechanism, metamaterial perfect absorbers with single and broad absorption peak were widely studied from radio frequency range to the optical realm^19^,^20^,^21^,^22^,^23^,^24^,^25^,^26^,^27^,^28^,^29^,^30^,^31^,^32^. Dual-band metamaterial perfect absorbers with two absorption bands were proposed subsequently because of their potential application areas such as detectors, transceiver system, and spectroscopic imagers^33^,^34^,^35^,^36^,^37^,^38^. However, all these dual-band metamaterial absorbers are based on metal “atoms” with different patterns. The anisotropy of metal “atoms” makes those absorbers sensitive to polarization and incident angles. Actually, dielectric “atoms” with high symmetry can also be used to construct metamaterial absorbers. When a plane wave is incident to a single isolated dielectric sphere with radius r and relative refractive index n, the scattered field can be decomposed into a multipole series^39^, with the 2^*m*^-pole term of the scattered electric field proportional to


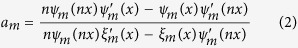


and the 2^*m*^-pole term of the scattered magnetic field proportional to


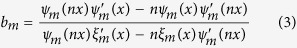


where *x* = *k*_0_*r*, *k*_0_ is the free-space wavenumber, *ψ*_*m*_ and *ξ*_*m*_ are the Riccati-Bessel functions. The scattering coefficient *a*_*m*_ and *b*_*m*_ are respectively related to the electric and magnetic responses of the sphere. Therefore, dielectric “atoms” can support a series of electric and magnetic resonance modes owing to multiple Mie resonances. Plenty of exotic electromagnetic properties have been demonstrated based on the Mie resonances of dielectric spheres^40^,^41^,^42^, cubes^43^,^44^ and rods^45^,^46^,^47^. In this work, we numerically and experimentally proposed a dual-band metamaterial perfect absorber with near unity absorptivity based on dielectric cubic “molecules” composed of four “atoms” of two different sizes.

## Results

### Design and numerical simulations

**Figure 1:**
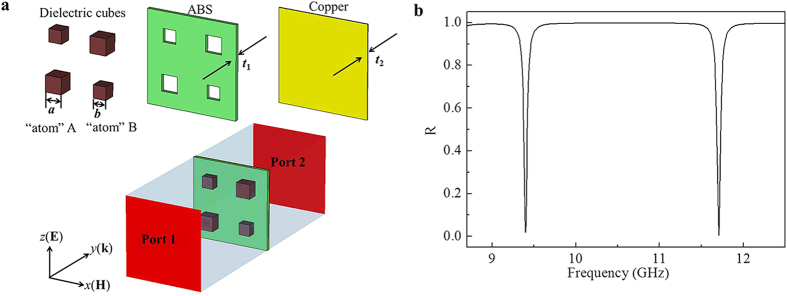
Unit cell characterization. (**a**) Schematic diagram of the unit cell of the dielectric “molecules” based dual-band metamaterial absorber. (**b**) The simulated reflection spectrum.

The unit cell of the dielectric “molecules” based dual-band metamaterial absorber is demonstrated in Fig. 1a. It consists of dielectric “atoms” with two different sizes (“atoms” A and “atoms” B) periodically embedded into a background matrix (acrylonitrile butadiene styrene: ABS) on the metallic ground plane. The unit cell of the metamaterial absorber has the dimensions, in millimeters, of: *a* = 1.8, *b* = 1.45, *p* = 10, *t*_1_ = 0.4, *t*_2_ = 0.1. The background matrix ABS with permittivity *ε*_1_ = 2.67 and loss tangent tan*δ*_1_ = 0.006 is used to fix the position of the dielectric “atoms”. The permittivity and loss tangent of the dielectric cubes are *ε*_2_ = 341, tan*δ*_2_ = 0.002, respectively. The metallic ground plane is made of copper with conductivity σ = 5.8 × 10^7^ s/m. Numerical simulations were performed using the finite-difference time domain (FDTD) method. The electromagnetic plane wave with incident angle *θ*, polarization angle *φ* was launched from Port 1 to the absorber sample along *y* direction. Periodic boundary conditions were set along the *x* and *z* axis. The simulated reflection spectrum (*θ* = 0, *φ* = 0) shown in Fig. 1b indicated that we achieved two reflection minimum with *R* = 2% at 9.4 GHz and *R* = 1% at 11.7 GHz. The transmission *T* is zero across the entire frequency range due to the metallic ground plane. Therefore, two absorption peaks with *A* = 98% at 9.4 GHz and *A* = 99% at 11.7 GHz were achieved according to *A* = 1-*R*-*T*.

**Figure 2:**
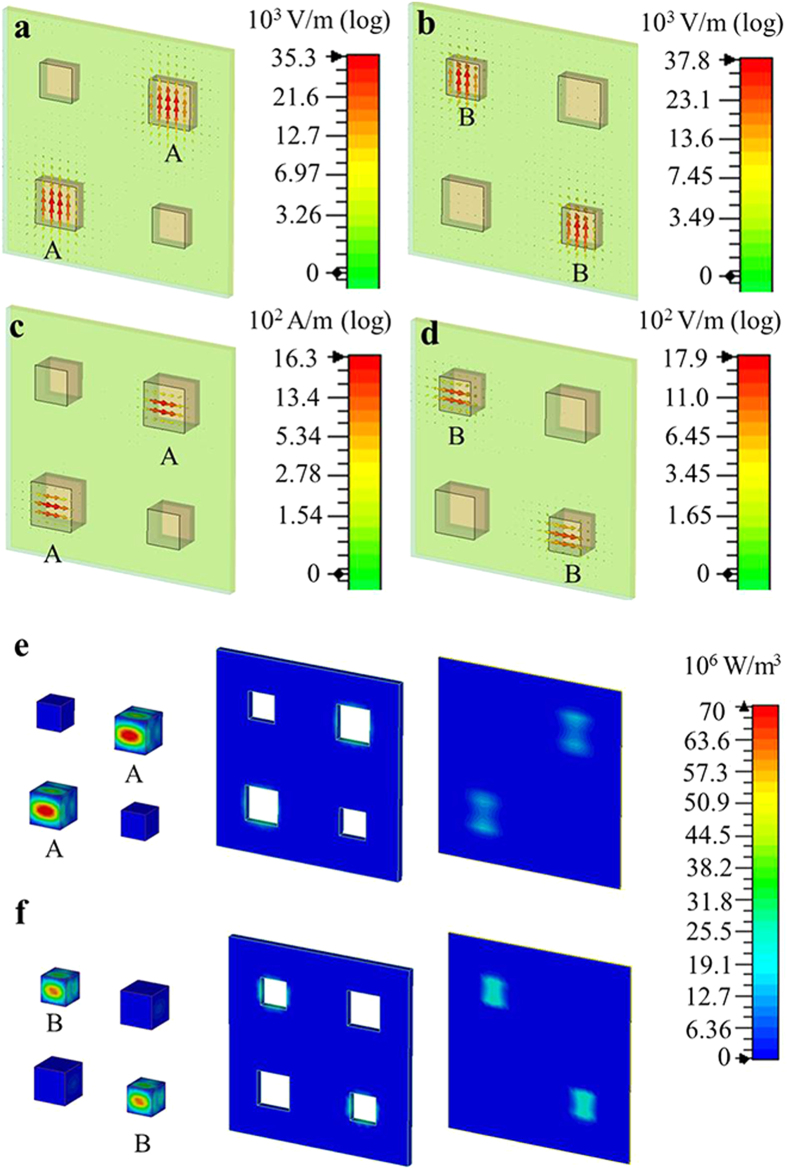
Numerical results of electromagnetic properties of the absorber. The electric field distributions in dielectric “atoms” at (**a**) 9.4 and (**b**) 11.7 GHz. The magnetic field distributions in dielectric “atoms” at (**c**) 9.4 and (**d**) 11.7 GHz. The power loss density in dielectric “atoms”, ABS, and copper at (**e**) 9.4 and (**f**) 11.7 GHz.

To explore how the two absorption bands were generated, we then simulated the electric and magnetic field distributions in the dielectric “atoms” at 9.4 and 11.7 GHz. As shown in Fig. 2a, there was a strong electric field distribution in “atoms” A with bigger size at lower frequency 9.4 GHz, which coupled strongly to the incident electric field leading to an intense electric resonance. At the same time, it is shown in Fig. 2c that there was also a strong magnetic field distributions in “atoms” A which generate a magnetic resonance at the same frequency. The similar electric and magnetic resonances were also found in “atoms” B with smaller size at 11.7 GHz, as demonstrated in Fig. 2b,d. The simultaneous resonances of electric permittivity *ε*(*ω*) and magnetic permeability *μ*(*ω*) made our metamaterial absorber impedance-matched to free space *ε*(*ω*) = *μ*(*ω*) at two absorption frequencies. The incident electromagnetic wave was trapped in the dielectric “atoms” without reflection *R*. The transmissivity *T* is zero across the entire frequency range due to the metallic ground plane. Therefore, two absorption peaks with near unity absorptivity *A* were achieved. The power loss density of this metamaterial absorber at 9.4 and 11.7 GHz in Fig. 2e,f indicated that the main loss was produced by the dielectric loss of the resonant dielectric “atoms”.

### Experimental results

**Figure 3:**
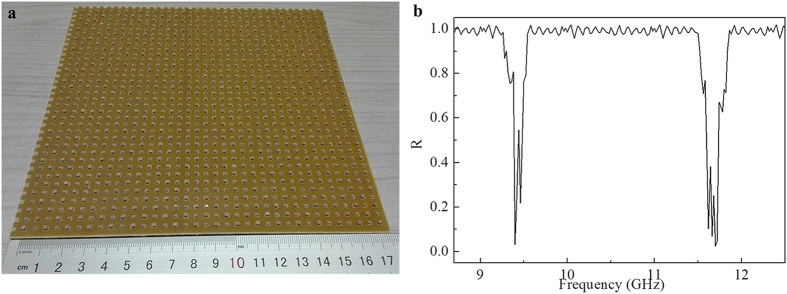
Experimental sample and characterization. (**a**) Dielectric “molecules” based dual-band metamaterial absorber sample. (**b**) The experimental reflection spectrum.

We built a dielectric “molecules” based dual-band metamaterial absorber sample according to the predesigned structure. The dielectric “atoms” was made by strontium titanate ceramic SrTiO_3_ (*ε*_2_ = 341, tan*δ*_2_ = 0.002), the absorber sample was composed of 289 artificial “molecules” (1156 artificial “atoms”) inserted into an ABS matrix on a metallic ground plane shown in Fig. 3a. The absorption performance was experimentally measured in free space by two linearly polarized antennas moving along the arc line. These two antennas were connected to a vector network analyzer (Agilent HP8720ES) to launch and receive electromagnetic waves. The experimental reflection spectrum shown in Fig. 3b demonstrated two reflection minimums with *R* = 3% at 9.4 GHz and *R* = 2.5% at 11.7 GHz. Therefore, two absorption peaks with *A* = 97% at 9.4 GHz and *A* = 97.5% at 11.7 GHz were experimentally achieved, which was in reasonable agreement with the simulation though the absorption peaks were lower than expected and there were some small split peaks due to fabrication imperfections.

## Discussion

**Figure 4:**
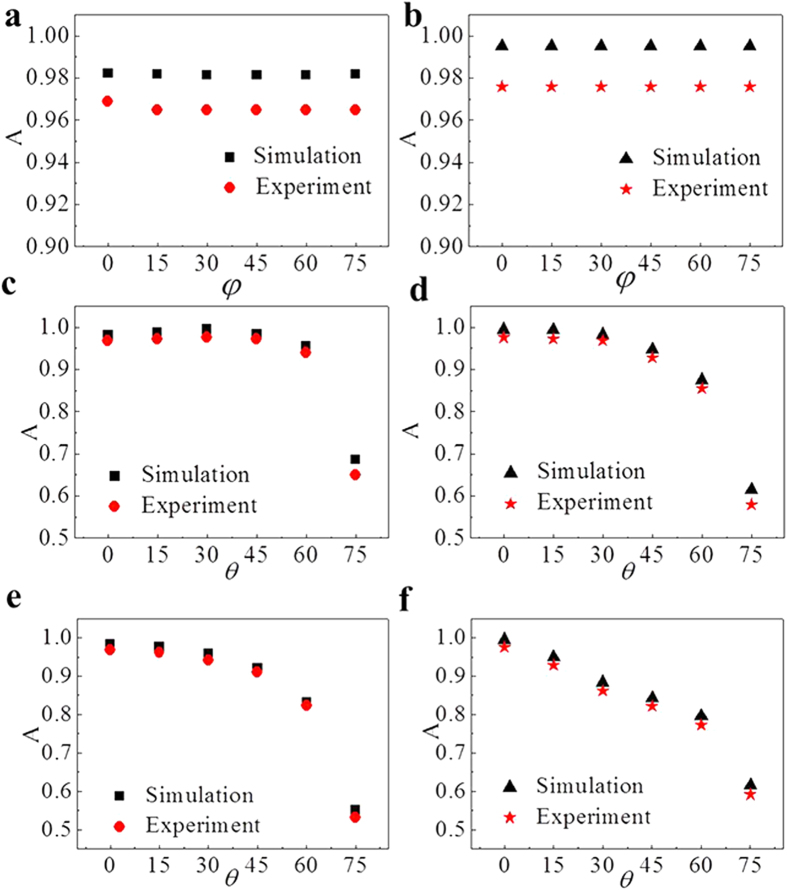
Absorptivity with polarization angle *φ,* and incident angle *θ* in TE and TM modes. Absorptivity with different polarization angle *φ* at (**a**) 9.4 and (**b**) 11.7 GHz, with different incident angle *θ* of the transverse electric (TE) wave at (**c**) 9.4 and (**d**) 11.7 GHz, with different incident angle *θ* of the transverse magnetic (TM) wave at (**e**) 9.4 and (**f**) 11.7 GHz.

Absorptivity with different polarization angle *φ,* and incident angle *θ* in transverse electric (TE) and transverse magnetic (TM) modes were further considered to evaluate the absorption properties. Figure 4a,b demonstrated the simulated and experimental absorptivity with different polarization angle *φ* at 9.4 and 11.7 GHz, respectively. It indicated that the two absorption peaks were independent on the polarization angle *φ* changing from 0 to 75 degrees. With TE incident wave, the absorptivity at 9.4 GHz was above 94% when the incident angle *θ* changed from 0 to 60 degrees. Then the absorptivity decreased dramatically from 94% to 65% as *θ* varied from 60 to 75 degrees shown in Fig. 4c. Figure 4d indicated that the absorptivity at 11.7 GHz was above 85% when the incident angle *θ* changed from 0 to 60 degrees. Then decreased from 85% to 60% as *θ* varied from 60 to 75 degrees. With TM incident wave, the absorptivity at 9.4 GHz decreased from 98% to 55% and the absorptivity at 11.7 GHz decreased from 99% to 60% when the incident angle *θ* changed from 0 to 75 degrees as shown in Fig. 4e,f. Simulated and experimental absorptivity was always above 78% when the incident angle below 60 degree in both TE and TM modes showing efficient function in wide-angle incidence.

In conclusion, a dual-band metamaterial perfect absorber based on artificial dielectric “molecules” was experimentally and numerically demonstrated. The metamaterial absorber consisted of a metallic ground plane and 289 dielectric cubic “molecules” (1156 dielectric “atoms”) embedded in ABS matrix. The dielectric “atoms” of different sizes coupled strongly to the incident electric and magnetic field at different frequencies leading to two absorption bands with simulated absorptivity of 98% and 99%, experimental absorptivity of 97% and 97.5% at 9.4 and 11.7 GHz. Numerical and experimental absorption spectra verified that the dule-band metamaterial absorber was polarization insensitive and could operate in wide-angle incidence.

## Methods

### Sample fabrication

High temperature solid-state reaction method was used to synthesize the ceramic material strontium titanate SrTiO_3._ We added 5% polyvinyl alcohol (PVA) to SrTiO_3_ powders with the mass ratio of 1:10 and mixed them homogeneously. The mixture was then uniaxially pressed into cylinders at 20 MPa, cold isostatically pressed at 200 MPa, and pressurelessly sintered at 873K for 2h and 1673 K for 4 h in air. Then we cut the ceramic cylinders into cubes with geometric parameters obtained from numerical simulations. We inserted dielectric cubes into the background matrix ABS and added a copper plate at the back to achieve the due-band dielectric metamaterial absorber.

## Additional Information

**How to cite this article**: Liu, X. *et al.* Dual band metamaterial perfect absorber based on artificial dielectric “molecules”. *Sci. Rep.* **6**, 28906; doi: 10.1038/srep28906 (2016).
